# Analgesic Effect of Cocaine and Lidocaine/Xylometazoline in Healthy Volunteers Undergoing Awake Nasal Intubation: A Randomized Controlled Crossover Trial

**DOI:** 10.1111/aas.70056

**Published:** 2025-05-10

**Authors:** Mo Haslund Larsen, Oscar Rosenkrantz, Rasmus Linnebjerg Knudsen, Rasmus Hesselfeldt, Ole Hilberg, Volkert Siersma, Johan Heiberg, Lars Simon Rasmussen, Dan Isbye

**Affiliations:** ^1^ Department of Anaesthesiology Centre of Head and Orthopaedics, Rigshospitalet, Copenhagen University Hospital Copenhagen Denmark; ^2^ Department of Clinical Epidemiology Aarhus University & Aarhus University Hospital Aarhus Denmark; ^3^ Department of Anaesthesiology and Intensive Care Nordsjællands Hospital Hillerød Denmark; ^4^ Institute for Regional Health Research, Sygehus Lillebælt Vejle Denmark; ^5^ The Research Unit for General Practice and Section of General Practice, Department of Public Health University of Copenhagen Copenhagen Denmark; ^6^ University of Copenhagen Copenhagen Denmark; ^7^ Danish Ministry of Defence, Personnel Agency Copenhagen Denmark; ^8^ Emergency Medical Services, Capital Region of Denmark Copenhagen Denmark

**Keywords:** cocaine, nasal, nasotracheal intubation, pain

## Abstract

**Background:**

Several drugs may be used to minimize pain during nasal intubation in awake patients. We hypothesized that the analgesic effect of cocaine would be at least as good (non‐inferior) as that of lidocaine with xylometazoline rated as maximum pain felt during awake nasal intubation of healthy volunteers.

**Methods:**

We conducted an outcome assessor blinded, randomized, triple crossover, non‐inferiority study following approval from the local research ethics committee and the national medicine agency. Healthy volunteers came for three visits and received 2 mL 4% cocaine, 0.5 mL 4% lidocaine + 1.5 mL 0.1% xylometazoline, and 2 mL 0.9% saline in random order prior to nasal insertion of an endotracheal tube. Maximum pain felt during insertion was evaluated on a visual analogue scale of 0–100 mm. The non‐inferiority margin was set to 11 mm on the visual analogue scale.

**Results:**

A total of 16 volunteers were enrolled, and 14 completed all three visits. Maximum pain felt during tube insertion was a median of 69 mm (interquartile range [IQR]: 56–73 mm) after cocaine, 60 mm (IQR: 50–76 mm) after lidocaine/xylometazoline, and 70 mm (IQR: 63–81 mm) after saline. The mean difference in maximum pain scores between cocaine and lidocaine/xylometazoline was 3.3 mm (95% confidence interval: −4.6 to 11.1; *p* = 0.40).

**Conclusion:**

We found no statistically significant difference in pain scores between cocaine and lidocaine/xylometazoline when administered prior to awake nasal intubation but cannot conclude that cocaine was non‐inferior to lidocaine/xylometazoline.

**Editorial Comment:**

Nasal intubation may be uncomfortable and can be complicated by epistaxis. Cocaine has both vasoconstrictive and analgesic properties and was compared with placebo and lidocaine/xylometazoline for awake intubation in healthy volunteers. The trial did not identify any clinically important differences between groups in terms of pain or serious adverse events. Differences were numerically small, and non‐inferiority between the active treatments was not demonstrated.

**Trial Registration:** Clinicaltrials.gov identifier: NCT06443255

## 1 Introduction

Nasotracheal intubation in awake patients requires topical analgesia and decongestion, and several drugs may be used in varying combinations and dosages [1, 2, 3, 4, 5, 6]. The 2020 Difficult Airway Society guidelines for awake tracheal intubation in adults advise against the use of cocaine for analgesia and vasoconstriction prior to awake nasotracheal intubation [7]. However, this recommendation is based on four case reports of cardiotoxicity [8, 9, 10, 11] and one study that did not demonstrate a difference in patient‐reported pain scores between co‐phenylcaine (a mixture of phenylephrine and lidocaine) and cocaine [12].

Cocaine is unique due to its combined analgesic and decongesting effects and is comparable to its alternatives regarding the prevention of epistaxis during nasotracheal intubation [13]. A combination of lidocaine and xylometazoline can also be used as lidocaine contributes with its analgesic effect while xylometazoline functions as a decongestant. Whether cocaine is comparable to lidocaine with xylometazoline in relieving pain during nasotracheal intubation is unknown. Therefore, we aimed to compare the analgesic effects of cocaine and lidocaine/xylometazoline when administered topically prior to awake nasal intubation of healthy volunteers.

We hypothesized that the analgesic effect of cocaine was at least as good (non‐inferior) as that of lidocaine/xylometazoline, rated as maximum pain felt during nasal insertion of an endotracheal tube.

## 2 Methods

### 2.1 Trial Design

This study was approved by the Danish Research Ethics Committee and Danish National Medicine Agency (CTIS registration number 2023‐506644‐17‐01) and written informed consent was obtained from all volunteers participating in the study. The trial was registered prior to patient enrollment at Clinicaltrials.gov (NCT06443255, Principal investigator: Mo Haslund Larsen, Date of registration: May 29, 2024). This manuscript adheres to the applicable CONSORT statement with the extension to cover randomized crossover trials [14].

We conducted a clinical, outcome assessor blinded, randomized, triple crossover, non‐inferiority study. The individual variation in pain perception and reporting rendered a crossover design the most appropriate, ensuring each participant functions as their own control. Participants had a wash‐out period of a minimum 7 days between their three visits to ensure elimination of all study drugs and sufficient recovery after any possible lacerations of the nasal mucosa. Participants were randomly allocated, so each study drug was received by one‐third of participants at their first visit in an attempt to distribute any potential bias from adaptation effects evenly.

The study was conducted in accordance with the prespecified trial protocol, with few exceptions: First, the planned secondary outcome of evaluating nasal decongestion by fiberoptic endoscopy was abandoned as the impact on the nasal mucosa compromised the evaluation of the primary outcome. Second, the endotracheal tube was not inserted to a depth of 10 cm if participants requested the tube to be removed prematurely due to pain. Third, due to staff shortages, the primary investigator was unblinded from the start of the trial in order to assist in randomization and preparation of study drugs. The primary investigator's participation in the intervention was limited to lubrication of the endotracheal tube and participation in obtaining outcomes was limited to conducting the acoustic rhinometry. Finally, due to a typing error the volumes of lidocaine and xylometazoline specified in the protocol were swapped in the written standard operating procedure for study drug preparation. This was the case for all participants and was discovered after conclusion of data collection.

### 2.2 Participants

Eligible participants were healthy volunteers, aged 18 years or older. Female participants of childbearing potential had to produce a negative urine human chorionic gonadotropin (hCG) test. Exclusion criteria were known nasal malformation, known coagulopathy, known hypertension, current antithrombotic treatment, self‐reported epistaxis occurring more than once a month, symptoms of a common cold within the past week of each visit, known hypersensitivity to any of the trial drugs, and narrow‐angle glaucoma.

Participants were recruited among medical students at Copenhagen University and received economical compensation for participation. The study location was the postoperative ward of the Ear‐, Nose‐, and Throat Department at Copenhagen University Hospital, Rigshospitalet, Copenhagen, Denmark.

Enrollment of new participants was terminated when complete data was collected on 12 participants. Participants already enrolled beyond this were completed.

### 2.3 Intervention

Study participants came for three visits where they received one of three study drugs: 2 mL 4% cocaine, 2 mL 0.9% saline, or 1.5 mL 0.1% xylometazoline immediately followed by 0.5 mL 4% lidocaine, the latter combination deviating from the 0.5 mL 0.1% xylometazoline and 1.5 mL 4% lidocaine specified in the protocol. Thus, all participants received a higher dose of the decongestive drug and a lower dose of the analgesic drug during the lidocaine/xylometazoline visit than planned in the study protocol. To ensure blinding, all interventions were prepared in two syringes: 0.5 mL in a 1 mL syringe and 1.5 mL in a 2 mL syringe, respectively. The study drugs were administered through the CE‐marked Mucosal Intranasal Rapid Atomization Device (MIRAD) (Marshall Products, Radstock, UK) by an anaesthesiologist who also performed the nasal intubation. Ten minutes after drug administration, nasal intubation was performed with a 6.0 mm tracheal tube (VentiSeal HVLP Cuffed Curved Nasal North ET Tube, Flexicare Medical Limited, Mountain Ash, UK) lubricated with a minimum of 2 mL Instillagel (20 mg/g lidocaine hydrochloride + 0.5 mg/g chlorhexidine gluconate). Instillagel was also liberally administered in the relevant nostril. The anesthesiological specialist advanced the tube to a maximum depth of 10 cm or chose to stop before this if loss of resistance was felt, indicating passage through the most narrow part of the nasal cavity.

The nostril chosen for passage of the tube was determined at the participant's first visit and maintained throughout the study. The participant was asked to evaluate which nostril allowed for better passage of air when closing off one nostril and performing a deep inspiration through the other, and vice versa. If there was no preference, the right nostril was chosen.

### 2.4 Outcomes

All outcomes were assessed at each of the participants' three visits. The primary outcome was maximum pain felt during insertion of the tube. This was reported by the participants immediately after removal of the tube on a visual analogue scale (VAS) ruler of 0–100 mm, where 0 mm was defined as no pain and 100 mm as the worst pain imaginable.

A secondary pain outcome was pain felt 1 min after removal of the tube, measured on the same VAS.

The decongestive properties of the study drugs were compared by quantifying the volume of the nasal cavity on the intervention side just prior to medicine administration and 2, 4, 6, 8, and 10 min after administration. This was assessed by acoustic rhinometry measured using the RhinoScan SRE2000 (Interacoustics A/S, Assens, Denmark). Volume of the nasal cavity and the narrowest cross‐sectional area was recorded for the outermost 54 mm of the nasal cavity.

Cocaine is associated with a risk of toxicity and the first signs of systemic uptake are hypertension, tachycardia, and hyperthermia. Patient safety measurements were therefore heart rate and arterial blood pressure prior to medicine administration and every minute for the first 5 min after drug administration, as well as temperature before medicine administration and 60 min after medicine administration.

### 2.5 Adverse Events and Serious Adverse Events

Adverse events and serious adverse events were defined prior to enrollment and consisted of headache, tachycardia, or hypertension requiring treatment, an unexplainable feeling of exaltation 5 min after drug administration, and tympanic temperature exceeding 39.0°C 60 min after drug administration. Nasal irritation and epistaxis commonly occur in patients after nasal intubation and were not considered adverse events. Serious adverse events were death, life‐threatening events, prolonged hospitalization, or disability. Adverse events and serious adverse events were evaluated after each visit by contacting the participants by telephone 7.5 h after drug administration. This time is equivalent to five half‐lives of cocaine, where the drug can be considered eliminated below a clinically relevant plasma concentration [15].

### 2.6 Randomization and Blinding

The order of the three treatments was one of three sequences following a Latin Square: (1) cocaine, followed by lidocaine/xylometazoline, then saline; or (2) lidocaine/xylometazoline, followed by saline, then cocaine; or (3) saline, followed by cocaine, then lidocaine/xylometazoline. The randomization list was generated in R (R Core Team, R Foundation for Statistical Computing, Vienna, Austria) from stacked blocks of randomized sequences of the Latin Square. This list, with a letter code representing the treatment sequences, was uploaded into the electronic data capturing system REDCap [16, 17] (Vanderbilt University, United States). An external data manager (S.K.) not involved in the study selected a random seed, executed the script, and uploaded the randomization list to REDCap. The primary investigator enrolled participants and entered inclusion data in REDCap.

After inclusion, the primary investigator conducted the randomization through the randomization module in REDCap and double‐checked it with a second unblinded research assistant. The letter code was translated to the study drug sequence using a conversion sheet only available to the primary investigator. Together, the unblinded primary investigator and unblinded research assistant subsequently prepared the chosen trial medication.

The participants and the anaesthetist were blinded to the allocation. The medications had identical labeling and packaging and were both colorless and odorless. The participant was asked not to reveal any unpleasant taste or sensation of numbness in the nose or throat upon administration.

The participants were unblinded after their final follow‐up, and the anaesthetist performing the intubation was blinded throughout the trial. The sponsor would only be unblinded in the occurrence of a serious adverse event. Blinding was assessed by asking the participant and the anesthetist performing the intubation which drug they thought was given.

### 2.7 Statistics

Baseline variables were summarized using medians with interquartile ranges (IQR) for continuous variables, and frequencies and percentages for categorical variables. The primary outcome of pain, measured on a VAS of 0–100 mm, was depicted in boxplots. The development of the secondary outcomes of nasal volume and cross‐sectional area measured by acoustic rhinometry was depicted in line plots with 95% confidence intervals (95% CI).

For each of the primary and secondary outcomes, the mean differences with corresponding 95% CI, between the three treatment modalities were assessed in mixed models, that is, linear regression models with a normally distributed error term using a participant identifier as a random effect. In these models, the visit order was additionally included as a fixed effect so as to adjust for possible adaptation effects.

The sample size was determined using a paired *t* test for non‐inferiority, assuming the order of the treatment modalities was immaterial and the true mean difference in VAS pain scores between the treatment modalities was nil. The non‐inferiority margin was based on a previous study that compared cocaine and co‐phenylcaine [12]. They included 25 participants and found a mean difference in pain ratings of 5.5 on the VAS. We decided to set our non‐inferiority margin at 11 mm, as that was considered to be a difference that would be clinically acceptable. We used a standard deviation of within‐person differences of 11 mm, as reported in a previous study [18]. Additionally, we assumed a correlation between paired measurements of 0.2. To obtain a power of 80% and a significance level of 5%, we required 12 participants to complete all visits. Additional participants were included in the event of drop‐outs to a total of no more than 25 included participants.

### 2.8 Statistical Software

Analyses were performed with SAS for Windows version 9.4 (SAS Institute Inc., Cary NC, USA). Figures were created using R version 4.3.0 (R Foundation for Statistical Computing, Vienna, Austria).

## 3 Results

### 3.1 Participant Demographics and Enrollment

**FIGURE 1:**
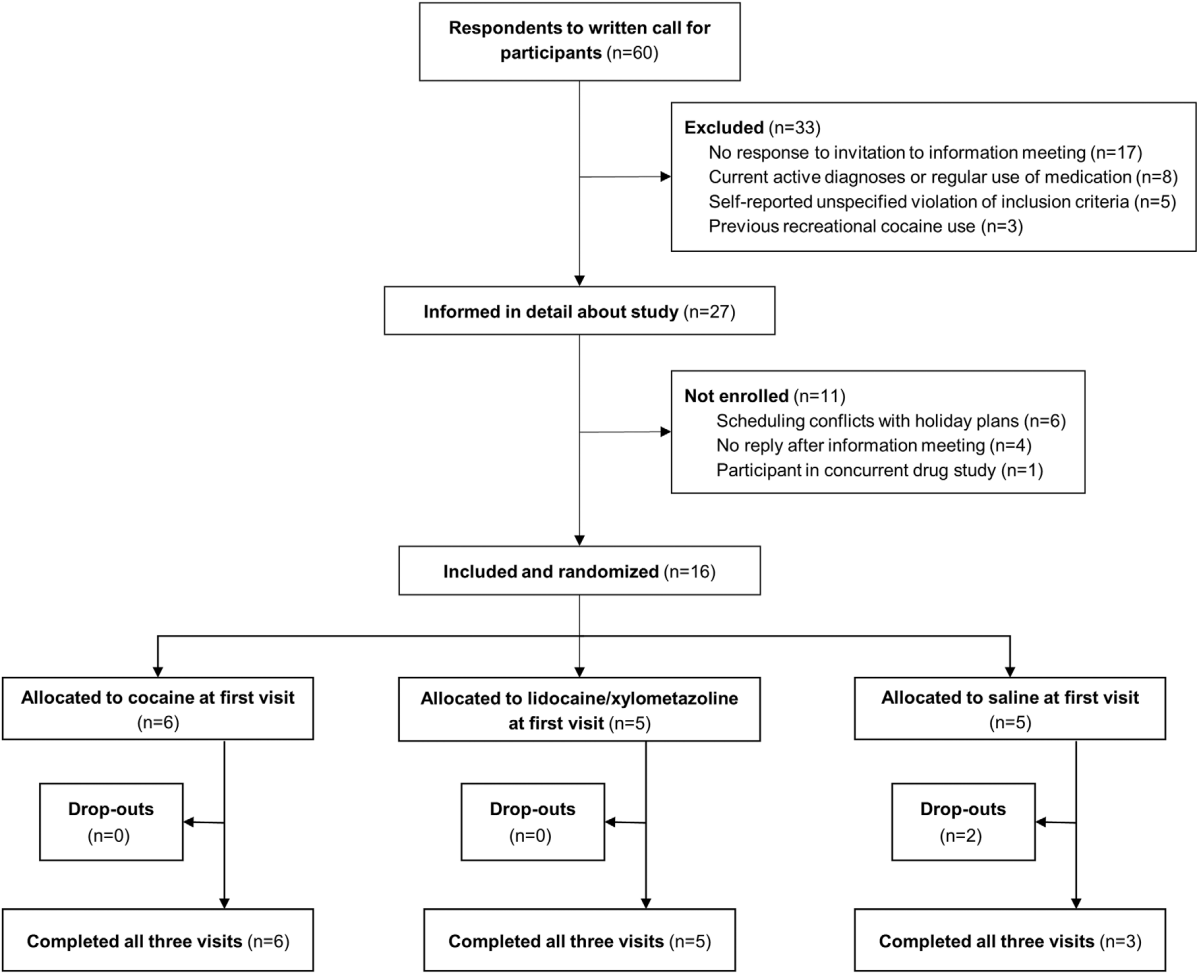
Participant enrollment flowchart. Healthy volunteers were randomized to cocaine, lidocaine/xylometazoline, or saline for their first visit. Two participants dropped out after their first visit.

We enrolled 16 participants between July 1, 2024 and July 26, 2024 and conducted the study between July 1, 2024 and August 27, 2024. Final follow‐up was completed 7.5 h after the last participant's last visit (Figure 1).

All data was included in the analyses. Baseline data were similar between the three randomization groups (Table 1).

**TABLE 1:** Characteristics of healthy volunteers randomized to either cocaine, lidocaine/xylometazoline, or saline at their first of three visits for nasal intubation.

	Cocaine (*n* = 6)	Lidocaine/xylometazoline (*n* = 5)	Saline (*n* = 5)
Age (yr), median [IQR]	23.5 [22.3 to 24.0]	25.0 [24.0 to 26.0]	23.0 [23.0 to 24.0]
Female sex, *n*	3 (50%)	4 (80%)	3 (60%)
Body mass index (kg/m^2^), median [IQR]	21.2 [20.1 to 22.9]	19.8 [19.1 to 20.4]	19.1 [18.2 to 19.2]

*Note:* Median values with interquartile range [25th to 75th percentile] for continuous variables and frequencies and percentages for categorical variables.

Abbreviations: IQR, interquartile range; yr, years.

### 3.2 Primary Outcome

**FIGURE 2:**
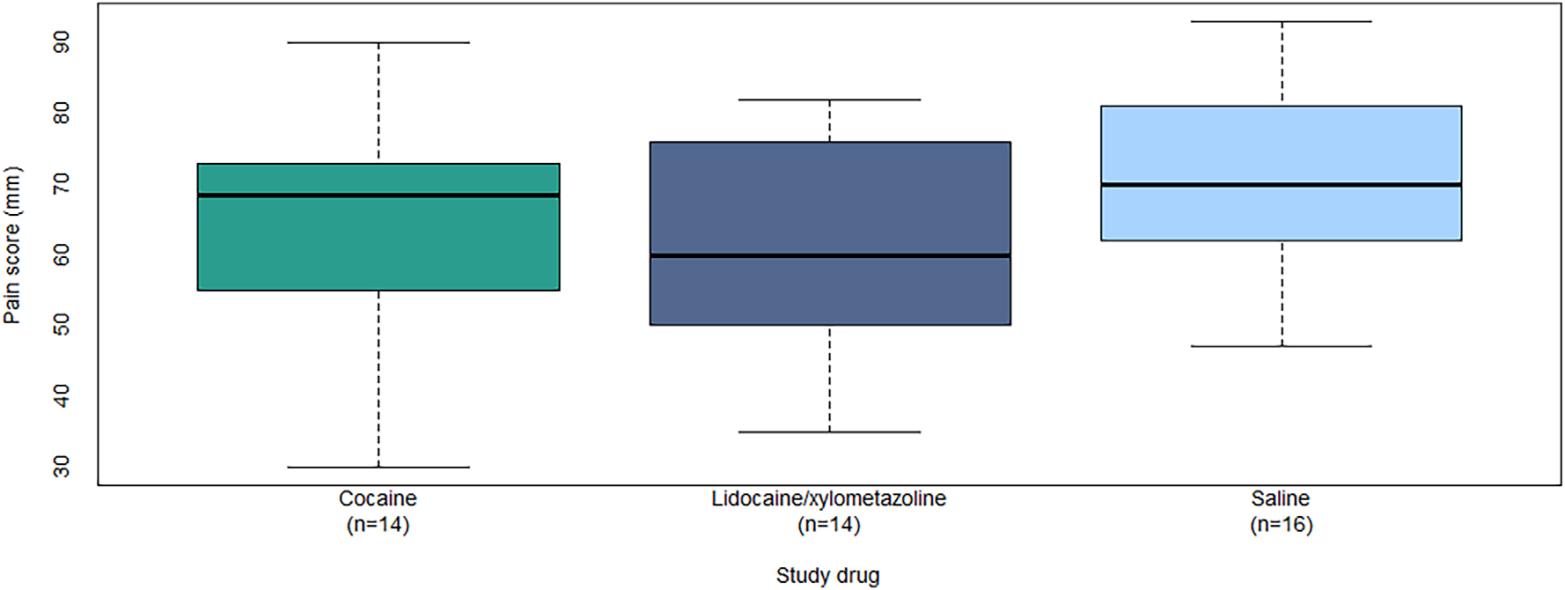
Boxplot of self‐reported maximum pain scores rated on a visual analogue scale of 0–100 mm for healthy volunteers for each intervention of cocaine, lidocaine/xylometazoline, and saline for nasal intubation.

The self‐reported maximum pain felt during tube insertion was a median of 69 mm (IQR: 56–73 mm) for cocaine, 60 mm (IQR: 50–76 mm) for lidocaine/xylometazoline, and 70 mm (IQR: 63–81 mm) for saline (Figure 2).

The mean difference in maximum pain scores between cocaine and lidocaine/xylometazoline was 3.3 mm (95% CI: −4.6 to 11.1) and not statistically significantly different (*p* = 0.40). Since the upper end of the 95% CI is greater than the non‐inferiority margin, we cannot conclude that cocaine is non‐inferior to lidocaine/xylometazoline.

There was no statistically significant difference in maximum pain score between the cocaine and saline (Table 2).

**TABLE 2:** Mean differences between the study drugs for primary and secondary outcomes.

	Cocaine—Lidocaine/Xylometazoline		Cocaine—Saline		Lidocaine/Xylometazoline—Saline	
	Mean difference (95% CI)	*p*	Mean difference (95% CI)	*p*	Mean difference (95% CI)	*p*
Primary outcome						
Pain during insertion (VAS 0–100 mm)	3.3 (−4.6 to 11.1)	0.40	−6.4 (−14.1 to 1.3)	0.10	−9.7 (−17.4 to −2.0)	0.016^a^
Secondary outcomes						
Pain after removal (VAS 0–100 mm)	2.8 (−7.3 to 13.0)	0.57	0.3 (−10.0 to 9.5)	0.96	−3.1 (−12.9 to 6.7)	0.52
Change in mean arterial blood pressure (0–5 min) (mmHg)	−1 (−6 to 3)	0.48	4 (−1 to 8)	0.083	5 (1 to 9)	0.018^a^
Change in heart rate (0–5 min) (bpm)	−1 (−7 to 5)	0.72	3 (−3 to 9)	0.33	4 (−2 to 10)	0.18
Change in nasal cavity volume (0–10 min) (cm^3^)	−0.30 (−1.43 to 0.82)	0.58	1.48 (0.38 to 2.57)	0.010	1.78 (0.68 to 2.88)	0.0027^a^
Change in minimal nasal cavity cross sectional area (0–10 min) (cm^2^)	−0.00 (−0.08 to 0.08)	0.95	0.08 (0.00 to 0.16)	0.051	0.07 (0.00 to 0.15)	0.059

Abbreviations: bpm, beats per minute; CI, confidence interval; mmHg, millimeters of mercury; VAS, visual analogue scale.

^a^ Statistically significant result.

### 3.3 Secondary Outcomes

The median pain scores after removal of the tube with interquartile ranges were 10 mm (IQR: 8–19 mm) for cocaine, 13 mm (IQR: 9–19 mm) for lidocaine/xylometazoline, and 15 mm (IQR: 12–26 mm) for saline. The mean difference in pain scores between cocaine and lidocaine/xylometazoline after removal of the tube was 2.8 mm (95% CI: −7.3 to 13.0) and not statistically significant (*p* = 0.57).

**FIGURE 3:**
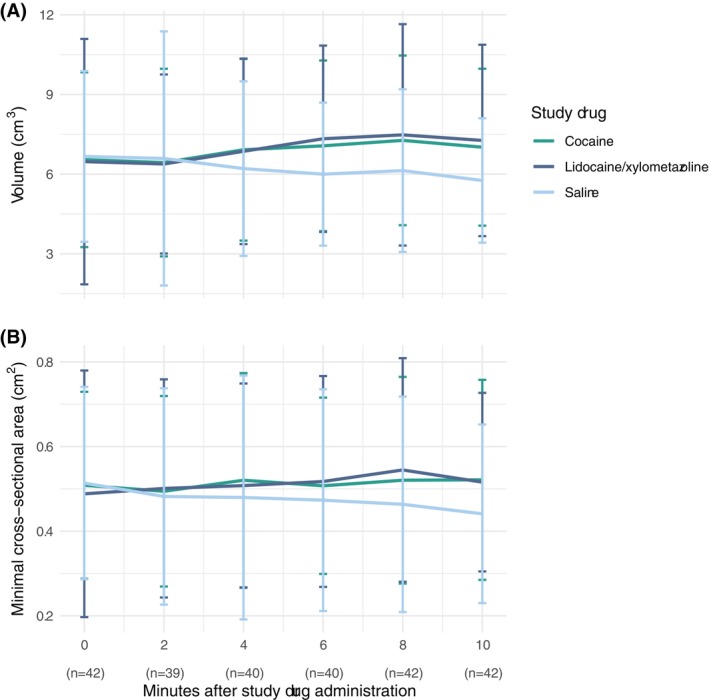
Mean nasal cavity volume in cm^3^ (a) and mean minimal nasal cavity cross‐sectional area in cm^2^ (b) in healthy volunteers randomized to cocaine, lidocaine/xylometazoline, and saline for nasal intubation. Measurements were done for the distal 54 mm of the nasal cavity on the side receiving the intervention. Vertical bars denote 95% confidence intervals. Each of the 16 participants had five attempted rhinometry measurements at each of their three visits (one visit for the two drop‐outs). The number of measurements (*n*) at each time point varied due to missing data.

We found a statistically significant larger increase in nasal cavity volume after both cocaine and lidocaine/xylometazoline compared to saline, but no difference between cocaine and lidocaine/xylometazoline (Figure 3 and Table 2). The same was found for minimum cross‐sectional area.

Insignificant changes were observed in mean arterial blood pressure and heart rate from just before to 5 min after drug administration. We found no evidence of differences between cocaine and saline mean arterial blood pressure: *p* = 0.083; heart rate: *p* = 0.33 (Table 2).

The participants and anesthetist correctly identified the blinded study drug 50% and 43% of the time for cocaine, 50% and 29% of the time for lidocaine/xylometazoline, and 69% and 63% of the time for saline, respectively.

No serious adverse events occurred during the study and no adverse events of hyperthermia, tachycardia requiring treatment, or hypertension requiring treatment were reported. Participants reported headaches in 10 out of 44 visits, distributed with five occurrences after cocaine (36%), three after lidocaine/xylometazoline (21%) and two after saline (13%). One participant experienced headaches after all three visits, two participants reported headaches after both cocaine and lidocaine/xylometazoline, and three participants reported one occurrence of headaches: two after cocaine and one after saline.

## 4 Discussion

In this study, we found no statistically significant difference in pain scores between cocaine and lidocaine/xylometazoline when administered prior to awake nasal intubation. As the confidence interval for the mean difference in maximum pain scores between cocaine and lidocaine/xylometazoline exceeds our non‐inferiority margin, we cannot conclude that cocaine is non‐inferior to lidocaine/xylometazoline in regard to analgesia during awake nasal intubation and consider our primary outcome inconclusive [19].

One previous study has investigated a similar research question [12]. Cara et al. compared cocaine and co‐phenylcaine sprays on participant‐reported pain on the VAS scale after the insertion of first a 7.0 mm nasopharyngeal airway and subsequently a 6.5 mm nasotracheal Portex tube. Similar to our study, they found no statistically significant differences between their two interventions. Another study [20] compared cocaine, lidocaine with oxymetazoline, and tetracaine with oxymetazoline for analgesia prior to nasal procedures and used the surrogate outcome measurement of pressure applied by monofilaments. They found tetracaine with oxymetazoline to be superior, but whether this can be extrapolated to apply for nasotracheal intubation is unknown.

Our study has several limitations. A possible source of bias was the unblinding of the primary investigator before study commencement. The primary investigator did not participate in the study drug administration, the insertion of the tube, or the pain evaluations, thus limiting the possible unintentional impact of this unblinding. The acoustic rhinometry was conducted by the primary investigator, but the standardization of the measurement procedure left little room for operator influence.

Our primary outcome was the maximum pain scores during intubation with a predefined non‐inferiority margin of 11 mm. We observed a mean difference of 3.3 mm in favor of lidocaine/xylometazoline with a 95% confidence interval of −4.6 to 11.1 mm. The study sample size was determined based on assumptions that may not have adequately accounted for variability in pain perception or procedural differences. The resulting wide confidence intervals reflect this limitation and underscore the challenge of drawing firm conclusions. Despite the crossover design enhancing statistical power, variability in self‐reported pain scores and individual tolerance thresholds remains a key consideration. Interestingly, no statistically significant difference in maximum pain was detected between cocaine and saline, but the 95% confidence interval for the difference was −14.1 to 1.3. This suggests that cocaine had some effect. However, lidocaine‐containing gel, which was used in all three groups, may have attenuated a clinical benefit.

The inadvertant exchange of volumes of lidocaine and xylometazoline resulted in a tripled dose of xylometazoline and one‐third dose of lidocaine compared to the intended. Whether the excessive decongestion compensated for the lesser analgesia is unknown, but we speculate that pain scores would have been lower after a higher lidocaine dose, thus increasing the difference in scores between cocaine and lidocaine/xylometazoline in favor of the latter. This would only further support the notion that cocaine cannot be considered non‐inferior to lidocaine/xylometazoline.

While the distribution of the data may be non‐normal, adequate inference assumes that the estimates are normally distributed. With 24 degrees of freedom for the mean difference estimates, we assessed this assumption as reasonable with reference to the central limit theorem and Figure 2 showing that the deviation of the data distribution within treatment modalities from the normal distribution is not extreme.

There was a risk of adaptation to the pain stimulus over the course of the three visits resulting in decreasing pain scores. This was accounted for by securing an equal amount of participants receiving each drug at each visit. Furthermore, we adjusted for the order in the analyses to counter any adaptation effects.

The length of insertion of the endotracheal tubes into the nasal cavity varied between participants and visits. However, we did not find this relevant, as pain, not insertion length, was our primary outcome. The participants were identically instructed at each visit that the goal was for a 10 cm passage, but they were at liberty to terminate the insertion when their personal pain threshold was met.

We conducted this study on healthy volunteers opposed to patients in need of awake fiberoptic nasotracheal intubation for surgery under general anaesthesia, as we wished to conduct a crossover study where each participant was their own control and had the same procedure completed multiple times. We attempted to ensure as uniform an intervention situation as possible: Throughout the study, participants sat in the same position in the same chair, and the insertion of the tube was performed by only one anesthesiologist, who regularly performs nasotracheal intubation. This ensured a uniform pressure and angling of the tube at all visits and eliminated inter‐operator variability. It could be argued that this decreases the external validity, but with a small sample size, we prioritized a standardized setting. It is common to sedate patients during awake intubations, but the role of sedatives was not evaluated in this study.

Acoustic rhinometry revealed a statistically significant increase in both nasal cavity volume and minimal cross sectional area over the course of 10 min after administration of cocaine or lidocaine/xylometazoline compared to saline. No significant differences in either volume nor minimal cross sectional area were found when comparing cocaine and lidocaine/xylometazoline.

Pain is a subjective parameter, and although the participants were blinded to their intervention, we could not blind their taste buds or limit their pharyngeal sensing. A salty taste or numbness of their throat may therefore have functioned as an unblinding by proxy and may have impacted their pain evaluations. In 50% of cases, participants correctly identified their study drug regarding both cocaine and lidocaine/xylometazoline, while saline was guessed correctly at 69% of visits. The salty taste of saline could have been a giveaway, and in hindsight, the use of sterile water as a placebo would have been preferable. Most likely, a correct identification of saline would underestimate the placebo effect. At each visit, participants were reminded not to comment on any taste or sensation as not to unintentionally reveal their allocation to the intubating anesthesiologist, who then could alter his procedure in response.

Systemic cocaine impacts the cardiovascular system by enhancing central sympathetic outflow, leading to peripheral vasoconstriction and an increased heart rate [21, 22]. The resulting hypertension and tachycardia are both dose‐dependent and immediate. Monitoring of blood pressure and heart rate revealed no significant differences between study drugs, suggesting minimal systemic absorption and minimizing concerns about cardiotoxicity in our participants. However, since our participants were young and healthy with no regular use of medications, these findings may not apply to populations of different demographics.

In conclusion, we found no statistically significant difference in pain scores between cocaine and lidocaine/xylometazoline when administered prior to awake nasal intubation but cannot conclude that cocaine was non‐inferior to lidocaine/xylometazoline. The results of this investigation cannot easily be used in decision making in clinical anesthesia as this was a study on unsedated healthy volunteers and the procedure differed somewhat from nasotracheal intubation. We intended to use drugs that are similar to those applied in the clinical setting but there is a large variation in the actual applications among clinicians.

## Author Contributions

The idea for this project was conceived by Lars Simon Rasmussen and Dan Isbye. Mo Haslund Larsen and Rasmus Hesselfeldt designed the work, and Mo Haslund Larsen and Rasmus Linnebjerg Knudsen prepared the study drugs and acquired the data. Johan Heiberg administered the study drugs and performed the intubations, and Ole Hilberg provided equipment and support for rhinometry. Mo Haslund Larsen, Oscar Rosenkrantz, and Volkert Siersma interpreted the data, and Mo Haslund Larsen wrote the first draft of this manuscript, which was critically revised and approved by all authors.

## Conflicts of Interest

The authors declare no conflicts of interest.

## Data Availability

The data that support the findings of this study are available from the corresponding author on reasonable request. The data are not publicly available due to privacy and ethical restrictions.
